# Exploring the associations of food and financial insecurity and food assistance with breastfeeding practices among first-time mothers

**DOI:** 10.1017/S1368980024001514

**Published:** 2024-09-06

**Authors:** Junia N de Brito, Jessica K Friedman, Sydney T Johnson, Jerica M Berge, Susan M Mason

**Affiliations:** 1 Department of Family Medicine and Community Health, University of Minnesota Medical School, Minneapolis, MN, USA; 2 Center for Care Delivery and Outcomes Research, Minneapolis VA Health Care System, Minneapolis, MN, USA; 3 Division of Epidemiology and Community Health, University of Minnesota School of Public Health, Minneapolis, MN, USA; 4 Department of Family Medicine, University of Colorado Anschutz Medical Campus, Aurora, CO, USA

**Keywords:** Breastfeeding, Women, Social determinants of health, Food insecurity, Food assistance

## Abstract

**Objective:**

Social determinants of health (SDoH), such as food and financial insecurity and food assistance, are potentially modifiable factors that may influence breastfeeding initiation and duration. Knowledge gaps exist regarding the relationship between these SDoH and infant feeding practices. We explored the relationships of food and financial insecurity and food assistance with the continuation of breastfeeding at four months postpartum among mothers and whether race and ethnicity modified these associations.

**Design:**

Mothers retrospectively reported food and financial insecurity and receipt of food assistance (e.g. Women, Infants and Children and Supplemental Nutrition Assistance Program) during pregnancy with their first child and infant feeding practices (exclusive/mostly breastfeeding *v*. exclusive/mostly formula feeding) following the birth of their first child. Sociodemographic-adjusted modified Poisson regressions estimated prevalence ratios and 95 % CI.

**Setting:**

Minneapolis-St. Paul, Minnesota.

**Participants:**

Mothers who participated in the Life-course Experiences And Pregnancy study (LEAP) (*n* 486).

**Results:**

Ten percent of mothers reported food insecurity, 43 % financial insecurity and 22 % food assistance during their pregnancies. At four months postpartum, 63 % exclusively/mostly breastfed and 37 % exclusively/mostly formula-fed. We found a lower adjusted prevalence of breastfeeding at four months postpartum for mothers who reported experiencing food insecurity (0·65; 0·43–0·98) and receiving food assistance (0·66; 0·94–0·88) relative to those who did not. For financial insecurity (aPR 0·92; 0·78, 1·08), adjusted estimates showed little evidence of an association.

**Conclusions:**

We found a lower level of breastfeeding among mothers experiencing food insecurity and using food assistance. Resources to support longer breastfeeding duration for mothers are needed. Moreover, facilitators, barriers and mechanisms of breastfeeding initiation and duration must be identified.

Breastfeeding offers numerous health benefits for birthing people (hereafter ‘mothers’) and children, yet these benefits are not equitably experienced by all mothers and children^(1)^. The social determinants of health (SDoH) are potential modifiable factors that influence breastfeeding initiation and duration^(2)^. Food and financial insecurity are complex SDoH factors that can potentially impact the ability of first-time mothers to breastfeed their infants. For example, food and/or financial insecurity may undermine access to nutritious and nourishing foods and/or force mothers to return to work shortly after giving birth, due to limited family leave policies at many US-based employers. Social welfare programs, such as the Special Supplemental Nutrition Program for Women, Infants and Children (WIC), may also play a significant role in influencing US mothers’ decisions to initiate and sustain breastfeeding. This program provides comprehensive nutrition and breastfeeding education to pregnant people and new mothers, which has the potential to encourage mothers to breastfeed. However, the provision of formula subsidies within WIC may inadvertently have the opposite effect by facilitating formula feeding instead^(3)^. The burgeoning literature examining whether food insecurity and access to food assistance programs in the US influence breastfeeding duration has demonstrated inconsistent findings^(4–6)^. In addition, much less is known about the relationship between financial insecurity and breastfeeding duration.

The purpose of this study was to explore the relationships of food and financial insecurity, as well as food assistance, with breastfeeding continuation at four months postpartum in a sample of first-time mothers. Examining these relationships at four months postpartum is critical. This period represents a pivotal time in their breastfeeding journey, as mothers face many important decisions, including introducing solid foods and navigating challenges related to returning to work while maintaining breastfeeding^(7)^. We further explored whether these relationships differed by race and ethnicity given existing disparities in breastfeeding prevalence^(8)^. Understanding these relationships will provide insights into whether interventions aimed at improving food and financial support could improve breastfeeding goals and reduce breastfeeding disparities, ultimately promoting equitable breastfeeding outcomes to improve the health of both mothers and infants.

## Methods

**Fig. 1:**
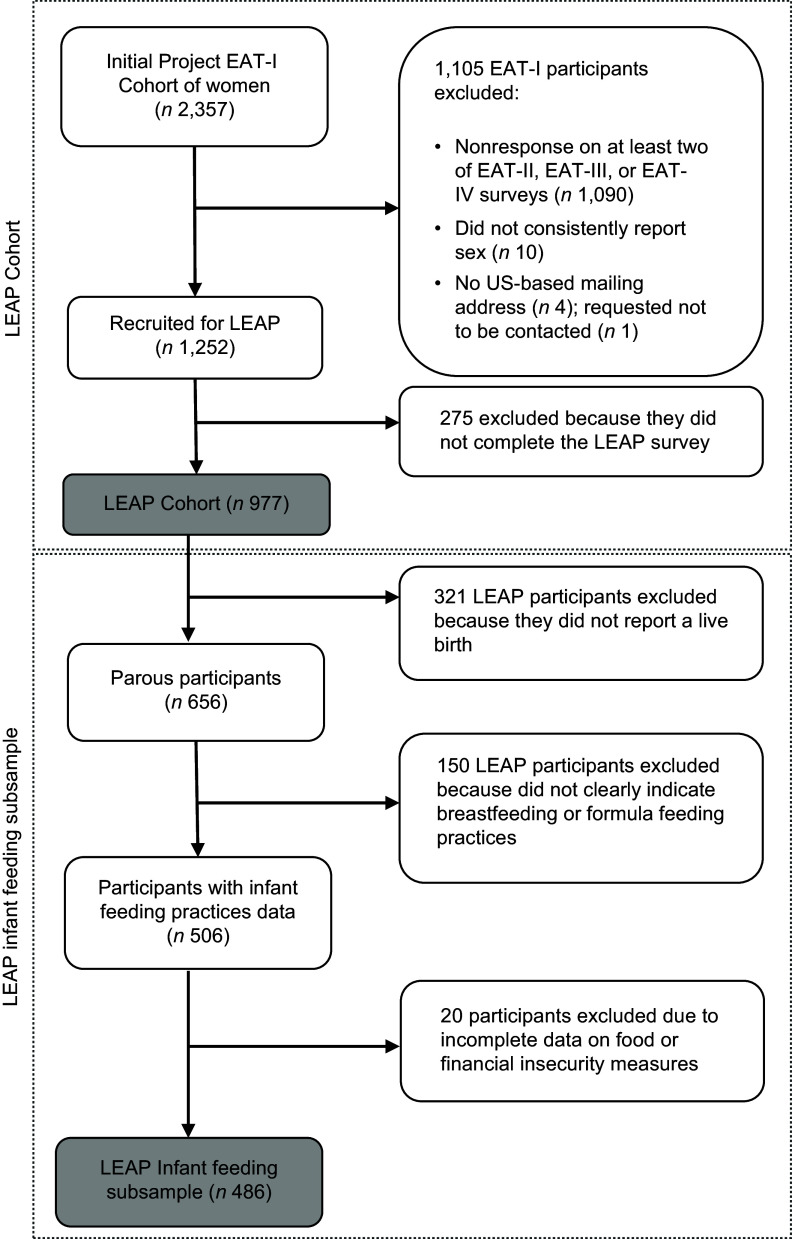
Flowchart of Project EAT, LEAP and LEAP subsample participants

Data are from the Life-course Experiences And Pregnancy (LEAP) study, a retrospective cohort of perinatal health among 977 women participating since adolescence in the Eating Activity in Teens and Young Adults (Project EAT), a longitudinal cohort study. Project EAT participants were recruited from the Minneapolis-St. Paul, Minnesota, public schools at ages 11–18 years from 1998–1999. Data were collected every five years, with the most recent EAT survey (EAT-IV) completed during 2014–2015. A flowchart (Fig. 1) details data collection, response rates and inclusion criteria. A detailed description of the Project EAT study aims, methods and main findings has been published elsewhere^(9–11)^. The analytic sample of the current study was restricted to parous people who self-identified as women or girls on the baseline EAT-I survey and who provided complete responses on food and financial security during pregnancy and infant feeding practices. In addition, we restricted to those who indicated exclusively or mostly feeding either formula or breastmilk at four months postpartum to ensure distinct comparison groups (*n* 486; 74 % of 656). The demographic distribution from the LEAP cohort, Project EAT-I and the current LEAP subsample is similar. All study protocols were approved by the University of Minnesota’s Institutional Review Board Human Subjects Committee, and electronic consent was obtained from all study participants.

### Measures

Survey items and coding decisions are presented in Table 1. The LEAP survey was designed to gather retrospective information from participants, specifically targeting non-overlapping timeframes. Specifically, food and financial insecurity and food assistance questions referred to *during pregnancy with their first child* period, and breastfeeding practices referred to *four-month postpartum* period. Infant feeding practices were assessed from a single adapted item indicating the relative proportion of breastmilk *v*. formula used for feeding within the first four months postpartum^(12,13)^. Participants who indicated using half formula/ half breastmilk (*n* 117) or did not provide an answer (*n* 1) were excluded from the analysis. This decision aimed to ensure the creation of distinct comparison groups, representing clear preferences for either breastfeeding or formula feeding. Food assistance, security and financial security were assessed using an adapted measure from Blumberg’s short form of the Household Food Security Scale^(14)^.

**Table 1:** Description of measures and coding decisions

Variable	Measure	Responses	Coding
Independent variable:			
Breastfeeding	What type of milk did you feed your first baby during his/her first 4 months?	1 (breastmilk only), 2 (formula only), 3 (mostly breastmilk), 4 (mostly formula), 5 (half formula/half breastmilk), 6 (prefer not to answer)	1 (1, 3) *v*. 0 (2, 4)
Dependent variables:			
Food insecurity	Food insecurity was assessed by four questions:	Answer options for question 1: 1 (Often true), 2 (somewhat true), 3 (never true)	Scores for questions 1a and 1b were dichotomised as 1 (1, 2) *v*. 0 (3) Total scores were derived for all five items and dichotomised as 1 (food insecure ≥ 3) *v*. 0 (food secure < 3
	1) While you were pregnant with your first child how often have the following statements been true for your household? 1a. The food we bought just didn’t last, and we didn’t have money to get more 1b. We couldn’t afford to eat balanced meals		
	2) While you were pregnant with your first child have you or other adults in your household ever cut the size of your meals or skipped meals because there wasn’t enough money for food?	Answer options for questions 2, 3 and 4: 1 (Yes)/ 0 (No)	
	3) While you were pregnant with your first child have you ever eaten less than you felt you should because there wasn’t enough money for food?		
	4) While you were pregnant with your first child have you ever been hungry but didn’t eat because there wasn’t enough money for food?		
Financial insecurity	While you were pregnant with your first child, how hard has it been for you to get by financially?	1 (not difficult at all), 2 (somewhat difficult), 3 (very difficult or can barely get by), 4 (extremely difficult or impossible)	1 (1) *v*. 0 (2, 3, 4)
Public food assistance	During 1^st^ pregnancy, have you received any of the following types of public assistance?	Check all that apply.	1 (any option checked) *v*. 0 (none checked)
Covariates	1) Food assistance: SNAP (Supplemental Nutrition Assistance Program), WIC (Women, Infants and Children), food from a food shelf/pantry		
Race and ethnicity	‘Do you think of yourself as	(1) White, (2) Black or African American, (3) Hispanic or Latino, (4) Asian American, (5) Native Hawaiian or Pacific Islander, (6) American Indian or Native American, or (7) Other’	1 (2, 3, 4, 5, 6, 7) *v*. 0 (1).
Maternal age at first pregnancy	Calculated based on date of first pregnancy resulting in a live birth minus participant birthdate	Date	Age (number)
Relationship status	Which of the following best describes your relationship status during your pregnancy with your first live birth?	1 (Married, living with spouse), 2 (unmarried, living with romantic partner), 3 (in a romantic relationship but not living together), 4 (not in a romantic relationship) 5 (other [open-ended])	0 (1) *v*. 1 (2, 3, 4, 5)

### Statistical analysis

In this secondary analysis of the LEAP study, we used modified Poisson regression models^(15)^ with robust SE to explore the associations between food and financial insecurity and food assistance, with breastfeeding continuation at four months postpartum. These models estimated prevalence ratios and 95 % CI. We also explored effect measure modification by self-reported race and ethnicity by adding interaction terms to all models. Models were adjusted for age at first birth, relationship status during pregnancy and self-reported race and ethnicity to account for historical social construction of race and ethnicity categories. The interpretation of the results was focused on the magnitude, direction and precision (95 % CI) of effect estimates rather than relying on significant/non-significant interpretations.

## Results

### Baseline characteristics of participants

Nearly two-thirds of mothers self-identified as White and reported being married or in a domestic partnership (Table 2). Approximately two-thirds of mothers exclusively or mostly breastfed their infants during the first four months compared to one-third of mothers who exclusively or mostly formula-fed their infants.

*Sociodemographic-adjusted associations of food insecurity, financial insecurity and food assistance during pregnancy with breastfeeding for the first 4 months postpartum*

**Table 2:** Participant characteristics during the first pregnancy

	Infant feeding practice					
	Total (*n* 486)	SD or %	Breastfed (*n* 316)	SD or %	Formula-Fed (*n* 170)	SD or %
Age m(sd)	27	5·2	29	4·6	25	5·4
*Race/ethnicity, n(%)*						
Asian	78	16 %	33	11 %	45	27 %
Black	37	8 %	14	5 %	23	14 %
Hispanic	18	4 %	11	4 %	7	4 %
Other race/ ethnicity	26	5 %	18	6 %	8	5 %
White	321	67 %	235	76 %	86	51 %
*Relationship status, n (%)*						
Married or domestic partnership	314	65 %	243	77 %	71	42 %
Other relationship status	172	35 %	73	23 %	99	58 %
*Economic hardship at time during first pregnancy, n (%)*						
* Receipt of food assistance*						
Yes	105	22 %	37	12 %	68	40 %
No	381	78 %	279	88 %	102	60 %
* Food insecurity*						
No food insecurity	436	90 %	300	95 %	136	80 %
Food insecurity	38	8 %	14	4 %	24	14 %
Hunger	12	2 %	2	1 %	10	6 %
* Difficulty getting by financially*						
Not at all difficult	277	57 %	210	66 %	67	39 %
Somewhat difficult	175	36 %	96	30 %	79	46 %
Very difficult or can barely get by	25	5 %	9	3 %	16	9 %
Extremely difficult	9	2 %	1	<1 %	8	5 %

Note: Numbers and % may not add up to 100 % due to missing observations.

Experiencing food insecurity during pregnancy was associated with 0·65 (95 % CI = 0·43, 0·98) times the prevalence of exclusively or mostly breastfeeding during the first four months postpartum compared to food security, after adjustments for sociodemographic covariates. Mothers who experienced financial insecurity had 0·92 (95 % CI = 0·78, 1·08) times the prevalence of exclusively or mostly breastfeeding during the first four months postpartum relative to mothers who were financially secure, after adjustments for sociodemographic factors. Receipt of food assistance, including accessing a food shelf or receiving benefits through safety net programs including WIC during pregnancy, was associated with 0·66 (95 % CI = 0·49, 0·88) times the prevalence of breastfeeding during the first four months postpartum, compared to no food assistance, after adjustments for sociodemographic covariates (Table 3).

**Table 3:** Associations of food and financial insecurity and food assistance with breastfeeding continuation at four months of age and effect measure modification by race and ethnicity

	Breastfeeding		*P* values for statistical interactions
	PR*	95 % CI	
Model 1 (unadjusted)			
Food insecurity	0·47	0·31, 0·70	*P* = 0·33
Financial insecurity	0·67	0·58, 0·78	*P* = 0·42
Food assistance	0·48	0·37, 0·63	*P* = 0·31
Model 2 (adjusted)			
Food insecurity	0·65	0·43, 0·98	*P* = 0·25
Financial insecurity	0·92	0·78, 1·08	*P* = 0·38
Food assistance	0·66	0·49, 0·88	*P* = 0·62

Model 2 was adjusted for maternal age, race/ethnicity and relationship status.

* Prevalence ratio.

### Effect measure modification by self-reported race and ethnicity

We did not find evidence of effect measure modification by race and ethnicity (P values for all statistical interactions were >0·2).

## Discussion

Our findings showed that mothers who reported experiencing food insecurity and those using food assistance programs during the prenatal period had a lower prevalence of breastfeeding at four months postpartum relative to mothers who did not report food insecurity or access to food assistance programs. Our results for financial assistance were close to the null. The point estimate for financial insecurity indicated a slightly lower prevalence of breastfeeding but 95 % CI were consistent with both a 22 percent lower likelihood of breastfeeding and an 8 percent higher likelihood of breastfeeding at four months after childbirth. Lastly, we did not find evidence for effect measure modification by race and ethnicity. These findings extend the literature documenting the impact of SDoH, such as food insecurity and access to food assistance programs on infant feeding practices.

Our findings of a negative association between food insecurity during pregnancy and breastfeeding are consistent with both observational and qualitative studies^(16,17)^. The premature discontinuation of breastfeeding among mothers living in food-insecure households reduces the likelihood of both mothers and infants experiencing the health-related benefits linked to breastfeeding^(18,19)^. For mothers, they are less likely to benefit from the added health gains that breastfeeding can promote, such as potential for a lower risk of developing hypertension, hyperlipidaemia, type 2 diabetes, different types of cancers (e.g. breast, ovarian, endometrial cancer) and CVD^(20)^. Infants are more likely to be deprived of key nutrients offered by breast milk, which play a vital role in strengthening their immune system and safeguarding them against a range of illnesses in the long term, such as asthma and certain infections^(21)^. Thus, addressing food insecurity, in addition to being important in and of itself, may support breastfeeding, promoting the health and well-being of both mothers and infants.

We also found a negative association between receiving food assistance during the prenatal period and breastfeeding at four months after childbirth. In the US, programs such as WIC provide nutrition and breastfeeding support and education to pregnant and postpartum people living in low-income households. While the WIC program has made notable strides in enhancing maternal and child health outcomes, observational studies have shown that mothers participating in WIC have a lower prevalence of breastfeeding continuation and are more likely to introduce formula earlier than current recommendations^(22,23)^. While the decision to stop breastfeeding is complex and multi-factorial, we note that the availability of free formula through WIC could be one of the many factors influencing the observed greater likelihood of formula feed in this sample. In the context of the US, paid parental or family leave is not federally mandated. In practice, this means that many mothers return to work shortly after giving birth due to economic pressures and concerns for job security. Compounded with workplace conditions that may foster an unsupportive atmosphere for breastfeeding (e.g. lack of lactation rooms, supportive culture and flexible work schedules) and childcare providers who might lack familiarity with handling breastmilk, these factors collectively pose an obstacle or impede mothers’ ability to sustain breastfeeding. Given these structural barriers, along with the pervasive availability of formula in the market influenced by the lobbying efforts of the milk industry, it is not surprising that many mothers who are relying on food assistance programs like WIC are more likely to introduce formula early in their infant’s feeding journey^(24)^. Thus, our results provide additional evidence for the need for future interventions targeting supports for longer breastfeeding duration and addressing the SDoH via policy change that provide and improve access to food and cash assistance programs, affordable and nutritious foods, federally mandated paid family leave and other resources to promote a conducive home and workplace environment for mothers who choose to breastfeed.

Lastly, we did not find evidence of an association between financial insecurity and breastfeeding. This finding is consistent with a prior observational study that investigated a number of indicators of socioeconomic status and reported that family income was not associated with breastfeeding^(25)^. More research is needed to further understand how various dimensions of financial insecurity, such as holding multiple jobs or facing unstable income, may impact breastfeeding practices. Nonetheless, while financial insecurity may not appear to be a direct barrier to breastfeeding in this sample, policies and programs aimed at reducing income inequality and increasing access to food assistance programs are important in supporting maternal and child health outcomes. To develop equitable breastfeeding promotion and support strategies for mothers, future research is needed to understand other potential barriers to breastfeeding (e.g. cultural, psychosocial, physiological and structural) in this population and elucidate the mechanisms through which food and financial insecurity and food assistance may adversely or positively affect breastfeeding initiation and duration.

Our findings should be interpreted in light of our study limitations. First, our study is limited by retrospective self-report measures, which are subject to recall and social desirability biases and could lead to measurement error. Second, our findings might not be generalizable to other geographical areas or to mothers of different cultural or socioeconomic backgrounds. More research is needed among racially, ethnically and socioeconomically diverse populations. Third, we only have data about infant feeding practices after the first live birth. We recommend future studies explore the consistency of our findings across subsequent live births.

## Conclusions

We found a lower prevalence of breastfeeding among mothers that reported experiencing food insecurity and using food assistance programs during the prenatal period. Policy-level changes tackling structural and social determinants of breastfeeding initiation and duration, including enforcing federally mandated paid family leave, workplace support for breastfeeding and improving access to affordable and nutritious foods are needed to support longer breastfeeding duration among mothers and advance breastfeeding equity.
